# Green Procurement Relationships Development under Carbon Emissions Regulations: A Bi-Level Programming Approach

**DOI:** 10.3390/ijerph15102183

**Published:** 2018-10-06

**Authors:** Xiao-Ying Bao, Lei Zhang

**Affiliations:** 1 College of Management Science, Chengdu University of Technology, Chengdu 610059, China; baoxiaoying205@163.com; 2 College of Business Administration, Zhejiang University of Finance and Economics, Hangzhou 310018, China; 3 School of Economics and Management, Fuzhou University, Fuzhou 350116, China

**Keywords:** supply chain management, optimization, bi-level programming, Stackelberg game, carbon emissions

## Abstract

A multi-period Stackelberg game is adopted to study a green procurement relationship between manufacturers and suppliers in a supply chain. The manufacturers are considered as leaders, while the suppliers are modelled as followers in this Stackelberg game. Accordingly, a mixed binary linear bi-level programming model is developed to elaborate the game in consideration of carbon tax scheme. The upper level (the leader) aims at selecting a proper number of suitable suppliers to provide heterogeneous raw materials at the lowest operational cost. The objective of the lower level (the follower) is to find optimal purchasing quantities of raw materials. In addition, two lemmas are introduced to transform the mixed linear bi-level programming model into a single level linear programming model. The numerical example illustrates that: (1) the manufacturer prefers to adopt the multiple sourcing strategy due to the flexibility; (2) keeping stable supplies and large order volumes could effectively reduce carbon emissions for the suppliers and make the supply chain greener.

## 1. Introduction

In recent years, pollution and extreme weather events such as the phenomenon of haze and light mist in China are seriously affecting human living conditions and agricultural production all over the world. Meanwhile, economic and social development is also influenced by the global climate change. Therefore, awareness of environmental protection is attracting considerable attentions from all sections of the community. As to the academic field, based on the traditional concept of supply chain management (SCM), green supply chain management (GSCM) has been proposed to deal with these comprehensive and complex problems from the operations management perspective. During the past decades, numbers of researchers explained the concept of GSCM from various angles, such as material management, procurement relation, inventory management, information mining, capital operation, ethical reason, reverse logistics, the triple bottom line (environmental, social, and economic), and ecological balance. In addition, different topics and applications in GSCM are also widely discussed [1,2,3,4,5,6,7].

Green procurement, as an important topic in GSCM, has always received much attention. In today’s increasingly competitive business environment and customer demand for eco-products, a growing number of international companies have realized the importance of global procurement with environmental concern. For instance, Li & Fung has established a global supply chain with over 15,000 suppliers in more than 40 economies [8]. Besides, Li & Fung has implemented a sustainable strategy to manage their environmental footprint and suppliers. Compared against a baseline in 2010, their carbon intensity was reduced by 23% in respect of Scopes 1 and 2 emissions [9]. IBM SCM offers a series of strategies to improve procurement effectiveness, such as supplier relationship management, and procurement one place strategy [10]. In addition, since 2010, IBM has established a series of requirements and measures for cooperation with environmentally and socially responsible suppliers, such as global requirements for waste processing, product end-of-life management, and, environmental evaluations of suppliers [11]. In the Assemble-To-Order environment, an assembler, like Toyota, plays a leadership role in its supply chain, while its suppliers follow the leader’s business actions. How to effectively coordinate relationships between leaders and followers? Such a phenomenon of cooperation and competition between buyers and suppliers can be perfectly described using a Stackelberg game. The objective of the leaders is to select appropriate suppliers while the suppliers (followers) need to decide their sales strategies and production (supply) plan to satisfy demand from downstream. The decision model, which is developed in this paper, not only considers traditional procurement and production criteria, but also includes environmental factors.

Given the serious environmental issues, carbon emissions regulations have been designed and implemented in different regions all over the world, especially carbon tax scheme in the European Union and China [12,13]. “Carbon tax schemes are a cost-effective means to curb GHG emissions from burning fossil fuels. This economic instrument can be regarded as one type of carbon pricing” [14]. Since the most of emissions are incurred during manufacturing processes by the manufacturer, thus, the production decisions of the manufacturer are influenced by carbon tax schemes. A natural question is: should the manufacturer source materials from a sustainable supplier with lower emissions and higher sales price or a general supplier with lower price with higher emissions? Therefore, how does the manufacturer makes reasonable decisions to maximize its profit under the carbon tax scheme by choosing multiple suppliers?

In this paper, to focus on these decision issues, we not only analyze the manufacturer’s decision issues but also consider suppliers’ operational decisions in a dynamic environment. The research questions can be summarized as follows: First, which sourcing strategy is the most profitable for the manufacturer and what is the according optimal sourcing quantity? Second, what role does each of carbon tax, the demand, the emissions rate of suppliers’ material play in the manufacturer’s optimal sourcing strategy? Therefore, to study these questions, the relationship between the manufacturer and its suppliers is described using a Stackelberg game and a bi-level programming model is formulated to assist the manufacturer making reasonable sourcing decisions.

The structure of the paper is organized as follows: in Section 2, the related literature is summarized. In Section 3, based on the bi-level programming approach, a multi-stage, multi-product, production-inventory-sourcing model is developed over a time horizon. In the Section 4, the numerical experiments are designed to solve the model and the possible sourcing strategies of the manufacturer are discussed. Section 5 concludes the paper.

## 2. Literature Review

With an increasing awareness of environmental protection, researchers have studied the effort of green procurement relationship on the philosophy of GSCM [15,16]. In the early days, green supplier ranking systems were developed for the selection of appropriate suppliers [17]. Subsequently, the analytical hierarchy process (AHP) model, which is integrated with green criteria, was developed to assess green suppliers [18]. Starting from the integrated multi-criteria decision making aspect, Kuo et al. [19] evaluated green suppliers for a camera manufacturer by the integrated models, including artificial neural networks (ANN)-analytical network process (ANP), and ANP-data envelopment analysis (DEA). The results showed that ANN-DEA has higher capabilities. Meanwhile, Zhou et al. [20] put forward a green supplier evaluation framework for the chemical industry. Similarly, Dou et al. [21] applied a grey ANP-based model to select green suppliers. In the situation of carbon management, He and Zhang [22] developed a hybrid model for supplier selection in low-carbon supply chain.

Different from multi-criteria decision making models, the following reviews briefly introduce the literature on green procurement relationship from the viewpoint of traditional modelling, such as game theory, statistical method, linear programming, dynamic programming. Hoetker et al. [23] analyzed three types of green procurement relationships, which were duration of buyer-supplier relationships, autonomy from customers, and links to prominent buyers. Two main results were obtained: both suppliers and buyers will benefit from the second relationship type; low and high modularity components benefit from the second and the third type, respectively. Xia et al. [24] studied a coordination issue of procurement a single product in a supply chain which is consists of multiple suppliers and multiple buyers. Considering the strong NP-hard nature of supplier scheduling problem, Selvarajah and Steiner [25] proposed an approximation algorithm, which also proved that the algorithm could find approximate solutions, for dealing with this issue.

Facing the complicated situation of asymmetric inventory information in green procurement, Zhang et al. [26] studied dynamic supplier contracts by analyzing a dynamic adverse-selection model. The results showed that batch-order contracts not only were the optimal choice in the infinite horizon, but also could minimize the information advantages of retailers. Meanwhile, Federgruen and Yang [27] analyzed an issue of procurement strategies with unreliable suppliers. They found that base-stock policy is no longer optimal and keeping reasonable number of suppliers depend on benchmark cost is the optimal choice. Driven by the financial incentive, Chen et al. [28] focused on the capacities allocation game between one supplier and two retailers. Based on the quantal response equilibrium (QRE), the model mainly illustrated two aspects: players are unperfected optimizers and there exists uncertainties of their opponents’ actions. Motivated by a spring up of supply chain intermediaries, Belavina and Girota [29] presented a novel stylized model to study direct and mediated sourcing. As for sourcing products from potential suppliers, an infinitely repeated game model was developed to solve this issue. Considering manufacturer cannot directly observe the effort of supplier which devoted during their cooperation process, Li et al. [30] emphasized on a long-term relationship between one manufacturer and two suppliers by presenting a repeated game model with an incentive scheme. Tang and Rai [31] studied the influence of two process capabilities on supplier relationships management. The results show that balancing factor is more effective than complementing factor, which can improve the competitive performance of a firm. Ji et al. [32] developed an evolutionary game model to evaluate suppliers who supply multiple raw materials. Guo et al. [33] discussed the sourcing police of a buyer choosing between responsible suppliers and risky suppliers

In order to analyze the impacts of carbon emissions regulations on green procurement, researchers have applied different types of the quantitative models. Choi [34] presented a supplier selection model under the carbon tax scheme, and developed the stochastic dynamic programming algorithm to select suppliers. Kumar et al. [35] suggested a comprehensive approach, which considered carbon footprints, for green supplier selection. The model encourages suppliers to toward green mode with reducing cost. Qi et al. [36] examined the pricing issue in a two-echelon supply chain with one supplier and two retailers under a carbon cap regulation. Considering the carbon information asymmetry, Yuan et al. [37] analyzed the optimal decisions of a low carbon supply chain including one retailer and on manufacturer under emissions trading scheme. Ma et al. [38] discussed the optimal procurement decision under carbon tax. They analyzed the impact of carbon tax on the optimal order quantity based on the dynamic programming model, and proposed the supplier evaluation procedure to select appropriate suppliers.

Most of the existing literature mainly studied how to evaluate green suppliers who only supply a single type of raw material. This paper studies green procurement relationships considering the multi-type raw materials procurement. In addition, the existing literature mostly adopted the single-period Stackelberg game models to study the green procurement relationship. This paper studies the multi-period case, and the multi-period mixed binary linear bi-level programming model is developed under a carbon tax scheme scenario. Accordingly, two lemmas are introduced, and the bi-level programming model is transformed into a single linear programming model, which can be easily solved. Moreover, numerical examples are provided to show the effectiveness of the model and analyze which sourcing strategy is selected under carbon tax scheme.

## 3. The Model

### 3.1. Problem Descriptions and Assumptions

In this section, we consider a supply chain in which manufacturers produce multiple products using multiples components from independent suppliers to meet its demand over a finite time horizon. This decision problem is formulated as a Stackelberg game in which the manufacturers are the leaders and the suppliers are the followers. The manufacturers first select appropriate suppliers. Then, to satisfy the manufacturers’ demand, each selected supplier makes production to minimize its total cost. Moreover, under the carbon tax scheme, the suppliers will pay an additional cost due to the carbon tax. Therefore, there exists a trade-off, that is, the manufacturers may source from a greener supplier with a lower emission rate and a higher cost or the manufacturers may select a more general supplier with a higher emissions rate and a lower cost. In practice, since the manufacturers can cooperate with diverse supplier in a loose supply market, each individual supplier can be mainly characterized from two aspects: cost and emissions rate of materials. Regarding suppliers are equivalent in operational aspects, such as marginal production, emissions rate, production capacity, etc. Thus, we assume each supplier cannot dominate each other, that is, one supplier is more “greener” and costlier than other suppliers.

**Figure 1:**
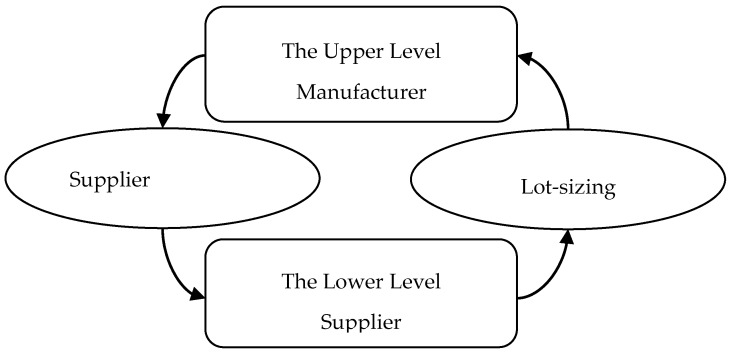
The procurement decision process.

In addition, we assume the full information structure of each supplier is known to the manufactures, that is, the any asymmetric information of each supplier can be reduced by the manufacturers before making production and sourcing decisions. Furthermore, it is assumed that the manufacturers are the risk-neutral cost minimizers. The structure of the above decision process is shown in Figure 1.

From Figure 1, the manufacturers need to jointly control production planning and make sourcing decision based on downstream sales information and daily production operations data. Then, the suppliers supply reasonable quantities considering its operations costs (i.e., production cost, emissions cost, and setup cost), production capacity, and inventory level. Therefore, we develop a mixed binary linear bi-level programming model to illustrate the relationships between the manufacturers and the suppliers. The parameters and decision variables used in the model are summarized in Table 1.

### 3.2. Manufacturer’s Decision (The Upper Level)

Under the carbon tax scheme, the goal of each manufacturer is to determine the sourcing and production decisions to satisfy the demands over a time horizon with minimum cost. Next, we aim to study a manufacturing system where the manufacturers produce multiple products using different types of components to meet the needs of its customers. As the leaders in the model, the manufacturers source components from independent suppliers to make final products based on the bill of materials. Adopting different types of components from independent suppliers with different costs and carbon tax of the manufacturers. Such a multi-period, multi-product, production planning and sourcing decision model, denoted as **PU**, can be developed as follows: 

**Problem PU**:

(1) $$\begin{array}{l} \min\;f_{1}=\sum_{i=1}^{l}\sum_{j=1}^{m}\sum_{k=1}^{n}\sum_{t=1}^{T}[E(p_{ik})x_{ijk}^{t}+PC_{jk}(x_{ijk}^{t}+I_{kj}^{t-1})CR_{jk}+[(I_{kj}^{t-1}+x_{ijk}^{t})(1-CR_{jk})]HC_{jk}+SC_{ij}^{t}S_{i}^{t}\\ s.t.\;\sum_{i=1}^{l}S_{i}^{t}\geq S^{t},\;t=1,2,\ldots ,T\\ \;\;\;I_{j}^{t-1}=0,\;t=1\\ \;\;\;S_{i}^{t}\in \left\{0,\;1\right\} \end{array}$$

The objective of **PU** is to minimize the total cost of the manufacturer with respect to the selection of the suppliers. The objective function consists of four parts, which are purchasing cost, production cost, holding cost, and setup cost. Here, the purchasing cost is equal to the unit price of different types of raw materials (*p_ik_*) times its purchased quantities (*x^t^_ijk_*). In the long-run, the prices of raw materials fluctuate between the highest and the lowest price, so, assuming price follows a uniform distribution the expected value is adopted to describe price. During the production process, raw materials purchased by manufacturers will be consumed and different types of raw materials have their own consumption ratios. The production cost and the holding cost are determined by the last period inventory level (*I^t−1^_kj_*) and reminder parts at *t* period. It is assumed the initial inventory levels of all kinds of raw material are zero. Moreover, the manufacturers will generate setup costs for selecting proper suppliers. In addition, the manufacturers should keep a reasonable number of suppliers in case of a shortage of raw materials to develop a relative safe and long term relationship with suppliers. The parameter *S^t^_i_* is a binary variable. *S^t^_i_* equals to one means the supplier *i* is selected in time period *t* and *S^t^_i_* equals to zero indicates the supplier *i* is not selected in time period *t*. The first constraint indicates that manufacturers can decide the number of suppliers to be selected.

### 3.3. Suppliers’ Decision (the Lower Level)

As a follower, the decision issue of a supplier is to make a reasonable production plan to match the demand of the manufacturer over a time horizon. A multi-period and multi-products production model, denoted as **PL**, can be formulated as follows:

Problem **PL**:

(2) $$\begin{array}{l} \min\;f_{2}=\sum_{i=1}^{l}\sum_{j=1}^{m}\sum_{k=1}^{n}\sum_{t=1}^{T}\left[g_{ik}x_{ijk}^{t}+TC_{ik}x_{ijk}^{t}+(PG_{ij}+TG_{ij})x_{ijk}^{t}GC_{ik}+R_{ik}x_{ijk}^{t}RC_{ik}+SC_{ik}^{t}S_{i}^{t}\right]\\ s.t.\;\sum_{i=1}^{l}\sum_{k=1}^{n}x_{ijk}^{t}\geq D_{jk}^{t},\;j=1,2,\ldots ,m\\ \;\;\;\sum_{i=1}^{l}\sum_{k=1}^{n}x_{ijk}^{t}\leq \sum_{i=1}^{l}C_{ik}S_{i}^{t},\;j=1,2,\ldots ,m\\ \;\;\;\sum_{i=1}^{l}\sum_{k=1}^{n}[p_{ik}-g_{ik}-TC_{ik}-(PG_{ij}+TG_{ij})GC-R_{ik}RC_{ik}]x_{ijk}^{t}\geq SC_{ik}^{t}S_{i}^{t},\;j=1,2,\ldots ,m\\ \;\;\;\sum_{i=1}^{l}\sum_{j=1}^{m}\sum_{k=1}^{n}x_{ijk}^{t}\geq 0,\;t=1,2,\ldots ,T \end{array}$$

The objective function of **PL** not only includes the traditional factors (production costs and transportation costs), but also focuses on the costs generated by greenhouse gas emissions. The production cost is determined by the unit raw material production cost (*g^t^_ik_*) and supply quantities (*x^t^_ijk_*). Similarly, the transportation cost equals to the unit transportation cost (*TC^t^_ik_*) times the ordered quantities (*x^t^_ijk_*). The carbon emissions cost includes two parts which are production (*PG^t^_ik_*) and transportation (*TG^t^_ik_*) carbon emissions. Besides, recycling cost is mainly decided by the recycling ratio (*R^t^_ik_*) of suppliers. The last part is the setup cost of suppliers. In Equation (2), the first constraint means total purchasing quantities of different raw materials should satisfy the demand quantities during each time period. As to the second one, the purchasing quantities cannot be larger than the production capacity of each selected supplier. The third constraint implies the profit of each supplier need to larger than its operational cost, otherwise, the supplier would lose money, which indicates that the supplier would not willing to cooperate with leaders. The last is non-negative constraint. Based on the above analysis, the mixed binary linear bi-level model not only considers the issue of green supplier selection, but also figures out optimal order quantities of different types of raw materials from selected suppliers. The complete model, denoted as **P1**, is developed as follows:

Problem **P1**:

(3) $$\begin{array}{l} \min\;f_{1}=\sum_{i=1}^{l}\sum_{j=1}^{m}\sum_{k=1}^{n}\sum_{t=1}^{T}[E(p_{ik})x_{ijk}^{t}+PC_{jk}(x_{ijk}^{t}+I_{kj}^{t-1})CR_{jk}+[(I_{kj}^{t-1}+x_{ijk}^{t})(1-CR_{jk})]HC_{jk}+SC_{ij}^{t}S_{i}^{t}\\ s.t.\;\sum_{i=1}^{l}S_{i}^{t}\geq S^{t},\;t=1,2,\ldots ,T\\ \;\;\;I_{j}^{t-1}=0,\;t=1\\ \;\;\;S_{i}^{t}\in \left\{0,\;1\right\}\\ \;\;\;x_{ijk}^{t}=\arg\min\;f_{2}=\sum_{i=1}^{l}\sum_{j=1}^{m}\sum_{k=1}^{n}\sum_{t=1}^{T}\left[g_{ik}x_{ijk}^{t}+TC_{ik}x_{ijk}^{t}+(PG_{ij}+TG_{ij})x_{ijk}^{t}GC+R_{ik}x_{ijk}^{t}RC_{ik}+SC_{ik}^{t}S_{i}^{t}\right] \end{array}$$

### 3.4. Model Solution

Problem P1 is a multi-stage, multi-product, production-inventory-sourcing bi-level model. Since the 1970s, bi-level programming problems have been extensively studied and bi-level programming still attracts researchers’ attention nowadays [39,40,41]. Dempe [40] has proved that a linear bi-level programming model is an NP hard problem. During the past decades, various methods and algorithms are suggested to solve bi-level programming problems. The Karush–Kuhn–Tucker (KKT) conditions are the most popular method to solve a bi-level model. This method aims at reducing the level of objective. However, this method increases the dimensions of variables and complexity of the model. Shih et al. [42] applied a bi-level programming model to optimize the subsidy rate for Taiwan’s glass recycling industry. By using the KKT conditions, a bi-level programming model was transformed into a mixed binary integer programming model. A harmonizing model with transfer tax (HMTT) was illustrated by Zhao et al. [43] using a bi-level programming model. According to the KKT conditions, the solution set of HMTT was observed to be non-empty, and a convergent algorithm was proposed to find solutions. The aforementioned papers showed that the procedure based on the KKT conditions can be applied to solve a bi-level programming model which has a single decision variable or a vector in the lower level. Once the lower level has more than one decision variable, complementary slackness conditions will generate a lot of constraints which increase the complexity of the model. Consequently, this section discusses another way to ameliorate one type of bi-level programming model which is the mixed binary linear bi-level programming model. Two Lemmas are provided as follows.

**Lemma** **1.** *Bi-level linear programming model can be transferred into a single level programming model by duality theorem.*

**Proof:**  Assuming the original bi-level linear programming model is shown as follows:

(4) $$\begin{array}{l} \underset{x\in X}{\min}\;F(x,y)\\ s.t.\left\{ \begin{array}{l} A_{1}x+B_{1}y\leq C_{1}\\ x\in \{0,1\}\\ \underset{y\in Y}{\min}\;D_{1}y_{1}+D_{2}y_{2}\\ s.t.\;\left\{ \begin{array}{l} A_{2}x+B_{2}y_{1}\leq C_{2}\\ A_{3}x+B_{3}y_{2}\geq C_{3}\\ y_{i}\geq 0,\;i=1,\;\;2 \end{array}\right. \end{array}\right. \end{array}$$

Assuming that x^*^ is the optimal solution of the upper level, then it will be a constant in the lower level programming model. The duality programming of the lower level is as follows:

(5) $$\begin{array}{l} \max\;(C_{2}-A_{2}x)\mu +(C_{2}-A_{2}x)\sigma \\ s.t.\left\{ \begin{array}{l} B_{2}\mu \leq D_{1}\\ B_{3}\sigma \leq D_{1}\\ \mu \leq 0\\ \sigma \geq 0 \end{array}\right. \end{array}$$

Based on the duality theory, there exists optimal *y*, *μ* and *σ* which satisfy:

(6) $$\left\{ \begin{array}{l} D_{1}y_{1}+D_{2}y_{2}-\;(C_{2}-A_{2}x)\mu -(C_{2}-A_{2}x)\sigma \geq 0\\ A_{2}x+B_{2}y_{1}\leq C_{2}\\ A_{3}x+B_{3}y_{2}\geq C_{3}\\ B_{2}\mu \leq D_{1}\\ B_{3}\sigma \leq D_{1}\\ \mu \leq 0\\ \sigma \geq 0\\ y_{i}\geq 0,\;i=1,\;\;2\\ x\in \{0,1\} \end{array}\right.\;$$

Then, the original bi-level model can be transferred into a single level programming model:

(7) $$\begin{array}{l} \underset{x\in X}{\min}\;F(x,y)\\ s.t.\left\{ \begin{array}{l} A_{1}x+B_{1}y\leq C_{1}\\ D_{1}y_{1}+D_{2}y_{2}-\;(C_{2}-A_{2}x)\mu -(C_{2}-A_{2}x)\sigma \geq 0\\ A_{2}x+B_{2}y_{1}\leq C_{2}\\ A_{3}x+B_{3}y_{2}\geq C_{3}\\ B_{2}\mu \leq D_{1}\\ B_{3}\sigma \leq D_{1}\\ \mu \leq 0\\ \sigma \geq 0\\ y_{i}\geq 0,\;i=1,\;\;2\\ x\in \{0,1\} \end{array}\right. \end{array}$$

The above transformation process introduces two dual variables (*μ* and σ) into the model. However, a nonlinear constraint also be introduced into the model. Thus, in order to further simplify the nonlinear constraint, an equivalence transformation be shown in the Lemma 2. □

**Lemma** **2.** *The nonlinear constraints can be equivalent transferred into linear constraints by M method in the mixed binary programming model.*

**Proof:**  According to the simplify process of the Lemma 1, there is a nonlinear constraint. Here, the nonlinear is generated by dual variables. In order to simplify this nonlinear constraint, two constant positive values *M*_1_ and *M*_2_ are introduced to relax it. The value of *x* is equal to 0 or 1. Thus, the two nonlinear variables can be replaced by:

(8) $$\begin{array}{l} m=A_{3}x\sigma ,\;\sigma \leq 0\\ n=A_{2}x\mu ,\;\mu \geq 0 \end{array}$$

Thus, two groups of constraints can be introduced to simplify these two types of nonlinear variables:

$$\left\{ \begin{array}{l} m\leq A_{2}\sigma -M_{1}x+M_{1}\\ m\geq A_{2}\sigma \\ m\geq -M_{1}x\\ m\leq 0 \end{array}\right.\;\mathrm{and}\;\left\{ \begin{array}{l} n\leq A_{3}\mu -M_{2}x+M_{2}\\ n\geq A_{3}\mu \\ n\leq M_{2}x\\ n\geq 0 \end{array}\right.\;$$

where *M*_1_ and *M*_2_ are large positive numbers. The above two groups provide the transformation procedure for dealing with both positive and negative nonlinear variables. □

According to Lemmas 1 and 2, Problem **P1** can be transformed into a single level linear programming model, which can be easily solved by linear optimization solvers, e.g., LINGO, MATLAB, or CPLEX.

## 4. Numerical Example

In this section, a numerical example is presented to describe green supplier selection and calculate purchasing quantities of multiple types of raw materials. The computational experiment is conducted on a dual processor Lenovo laptop working at 2.40 GHz and equipped with 4G memory (Lenovo, Beijing, China).

### 4.1. Parameters Setting

In the numerical example, we assume that a manufacturer purchases two types of raw materials (R1 and R2) from five suppliers. The parameters of the suppliers are shown in Table 2. Moreover, Table 3 describes the operation data of the manufacturer. Demand of raw materials R1 and R2 follows the Poisson distribution with *λ*_1_ = 700 and *λ*_2_ = 550. The setup cost of a manufacturer is 400 for the selection each supplier.

In addition, the demands of the raw materials (R1 and R2) in different periods are randomly generated by MATLAB 2012b (The MathWorks, Inc, Natick, MA, USA), and shown in Table 4.

### 4.2. Results Analysis and Discussion

Combining the data in above two tables and the simplified processes which are illustrated in Section 3.4, the simplified model can be solved by using LINGO 14.0 (LINDO Systems, Inc, Chicago, IL, USA). The inventory levels and current purchasing quantities are shown in Table 5, and the optimal purchasing quantities of selected suppliers are shown in Table 6.

From Table 5 and Table 6, we can see that different suppliers will be selected to supply two types of raw materials to the manufacturer. Moreover, the optimal purchasing quantities and the inventory levels are calculated, respectively. In practice, there are three possible sourcing strategies for the manufacturer, including the risky sourcing strategy, multiple sourcing strategy, and environmental responsibility sourcing strategy. In risky sourcing strategy, the manufacturer only sources higher emissions materials with the lower cost from the traditional suppliers. However, the manufacturer will pay a much higher carbon tax. Rather than adopt the risky sourcing strategy, the manufacturer could also cooperate with multiple types of suppliers to control its amount of emissions during the production processes. During the finite time horizon, making flexible adjustment of the sourcing profile for the manufacturer based on the carbon tax in each period may be profitable. In environmental responsibility sourcing strategy, the manufacturer only cooperates with the sustainable suppliers with higher cost. The lower carbon tax can also be used to make up for the expensive sourcing cost of the materials with lower emission rates. In addition, since an increasing customer demand for the sustainably produced products, and part of customers are willing to pay a premium, therefore, this sourcing strategy has been adopted in some companies, as aforementioned in Introduction, IBM and Li&Fung have implemented the environmental sourcing strategy to manage their suppliers and the carbon footprint of their supply chain.

From Table 6, the manufacturer prefers to adopt the multiple sourcing strategy since this strategy can flexibly balance the profit and the amount of emissions. Taking purchasing quantities of raw material 2 as an analysis target, supply quantities from supplier 2 and 4 accounted for nearly one hundred percent of the total demand. The sales price, production cost, transportation cost, and recycling cost are key factors which mainly influence market business volume share of suppliers. By calculation optimal solutions and analyzing purchasing parameters, although supplier 2 sells raw materials with higher price, supplier 2 has higher recycling ratio and lower emission ratio. Supplier 4 owns the characteristic of the lower emission ratio and reasonable production cost. Besides, results also indicate that supplier 2 and supplier 4 have the highest level of clean production technology skills. Their production and supply capability are tending to stability. They are key suppliers to the manufacturer. In terms of raw material 1, supplier 1 and 4 cooperate with the manufacturer with their maximum production capabilities, which means their cooperation relationship trends are stable. However, supplier 3 and 5 will become key suppliers of the manufacturer when the market demands increase suddenly.

**Figure 2:**
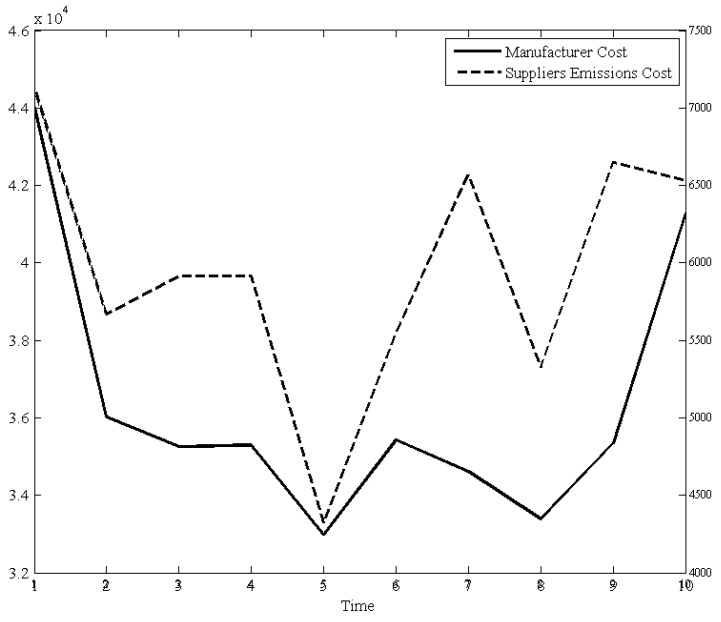
The affection of manufacturer cost and emissions cost of suppliers.

Figure 2 shows that the variance between the operational cost of the manufacturer and the emission cost of suppliers. It can be clearly find that these two sets of data reflect a sensitive relationship. During procurement periods from the second to the ninth period, emissions cost of suppliers (dashed line) fluctuate widely while the cost of the manufacturer (solid line) with a slight fluctuation. However, the operational cost of the manufacturer is mainly determined by its order quantities. This indicates that the periodic wide fluctuation of procurement will increase the emissions cost of suppliers, in other words, keeping stability procurement quantities could effectively draw down the carbon emissions cost of the upstream of a company.

**Figure 3:**
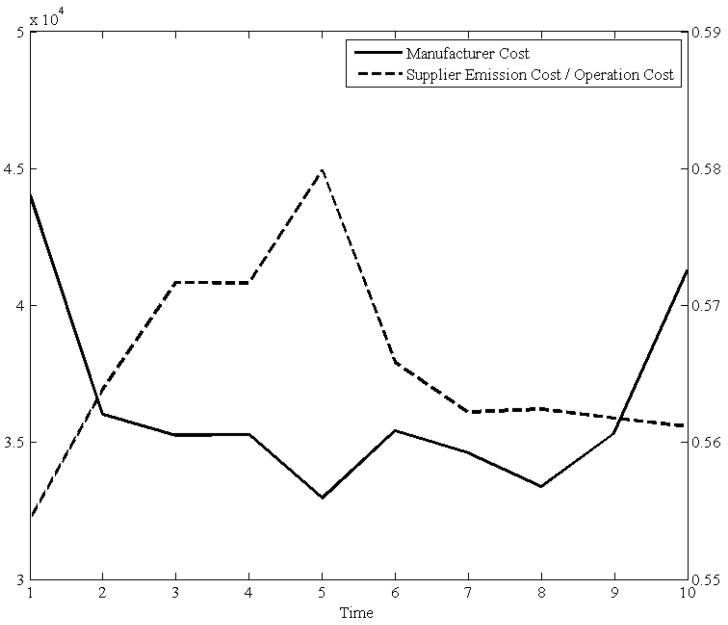
The affection of manufacturer cost and the ratio of supplier emissions.

In the Figure 3, the dashed line means the ratio (percentage) of suppliers’ emissions cost over its total operation cost in each period. From another aspect, Figure 3 illustrates that the lower the ratio (in periods 1 and 10) faces a large scale of procurement quantities. From the second period to the ninth period, lower procurement quantities with relative higher emission ratio of suppliers. This means large scale procurement could effectively maintain a relative lower emission ratio. However, large scale procurement also could increase the holding cost of a manufacturer. Therefore, the procurement policy designed by manager directly determined the green degree of the upstream of a supply chain.

According to the numerical analysis, three managerial implications can be summarized:

(i) The manufacturer should keep watch on the suppliers who supply their maximum supply capabilities. The bargaining powers of the suppliers from alliances will be enhanced. Then, the suppliers could bid up the price higher than the market can bear to make profits. Meanwhile, the raw material inventory level of the manufacturer will increase, which will add to the inventory cost and deprive the manufacturer of benefits. This sort of behavior also could lead to the fluctuation of purchasing quantities, which goes against drawing down the carbon emissions cost of the upstream of the manufacturer.

(ii) The manufacturers should also pay more attention to those suppliers whose supply quantities have the same floating trend with demands. It is because that they directly influenced the total quantities of the order. Thus, it is necessary to maintain a long-term relationship with this type of suppliers.

(iii) Based on the good cooperative relationships between the supplier and the manufacturer, maintenance large scale and stability procurement quantities could effectively improve the green degree of the upstream of the manufacturer.

## 5. Conclusions

Driven by green supply chain management and the green purchasing strategy, this paper adopts the Stackelberg game concept to study the procurement relationships between manufacturers (leaders) and suppliers (followers). The objective of leaders is to select a proper supplier to satisfy their demands, while the followers aim to determine the optimal order quantities. In order to fully reflect the game process between leaders and followers, a mixed binary linear bi-level programming model is developed. As to the upper level, the leader should determine the number of selected suppliers. According to optimal results of the upper level, the lower level will calculate optimal purchasing quantities which are supported by production capacity, recycling costs, carbon emissions cost, etc. In addition, two lemmas were introduced to be used to transfer a mixed binary linear bi-level programming model into a single level linear programming model.

The numerical example indicates that sales and environmental strategies play dominant roles which could help suppliers to enlarge their business volume. In addition, the numerical example not only proves that the two models can solve multi-type raw material purchasing issues, but also suggests that maintaining stability and large scale procurement quantities could effectively draw down the carbon emissions cost and improve the green degree of the upstream of a supply chain.

The limitations of this research are as follows: first, types of transportation are not discussed in detail, and different means of delivery have different emissions factor. Secondly, the decision process only considers a carbon tax scheme. In practice, carbon cap schemes and carbon trade schemes should be considered in green procurement. Thirdly, the decision process is an iterative process. We ignore the effect of repeated games in the multi-period Stackelberg game. Thus, the scope of the future research can start from the limitations presented above.

## Figures and Tables

**Table 1:** Notation.

*i*	The number of suppliers, *i* = 1, 2, 3,…, *l*
*j*	The number of manufacturers, *j* = 1, 2, 3,…, *m*
*k*	The type of raw materials, *k* = 1, 2, 3,…, *n*
*t*	The time period, *t* = 1, 2, 3,…, *T*
*p_ik_*	The unit sales price of raw material *k* from a supplier *i*
*g_ik_*	The unit production cost of raw material *k* from a supplier *i*
*TC_ik_*	The unit transportation cost of raw material *k* from a supplier *i*
*GC_ik_*	The carbon tax of unit raw material *k* from a supplier *i*
*RC_ik_*	The unit recycling cost of raw material *k* from a supplier *i*
*SC_ik_*	The setup cost of production raw material *k* from a supplier *i*
*C_ik_*	The production capacity of raw material *k* from a supplier *i*
*PG_ik_*	The carbon emission ratio of production unit raw material *k* from a supplier *i*
*TG_ik_*	The carbon emission ratio of transportation unit raw material *k* from a supplier *i*
*R_ik_*	The unit recycling ratio of raw material *k* from a supplier *i*
*HC_jk_*	The holding cost of raw material *k* from a manufacturer *j*
*PC_jk_*	The production cost of raw material *k* from a manufacturer *j*
*SC_ij_*	The setup cost of a manufacturer *j* selects a supplier *i*
*CR_jk_*	The consumption ratio of raw material *k* from manufacturer *j*
*I^t^_kj_*	In period *t*, the inventory level of raw material *k* from manufacturer *j*
*D^t^_jk_*	In period *t*, the demand of raw material *k* from manufacturer *j*
*S^t^_j_*	In period *t*, the supplier *i* is selected or not
*S^t^*	In period *t*, the minimum number of suppliers should be selected
*x^t^_ijk_*	In period *t*, the manufacturer *j*’s purchasing quantities of raw material *k* from a supplier *i*

**Table 2:** The parameters of the suppliers.

Supplier	Material Type	*p_ik_*	*g_ik_*	*TC_ik_*	*PG_ik_*	*TG_ik_*	*GC_ik_*	*R_ik_*	*RC_ik_*	*SC_ik_*	*C_ik_*
S1	R1	[30,36]	4	0.03	0.4	0.2	10	0.2	3	100	200
	R2	[20,30]	7	0.03	0.4	0.2	10	0.2	3	100	250
S2	R1	[45,55]	7	0.02	0.2	0.3	10	0.25	4	200	300
	R2	[20,24]	6	0.02	0.2	0.3	10	0.25	3	200	280
S3	R1	[35,45]	5	0.025	0.3	0.15	10	0.2	2	150	150
	R2	[23,29]	6	0.025	0.3	0.15	10	0.2	4	150	320
S4	R1	[32,44]	3	0.035	0.25	0.25	10	0.15	5	120	160
	R2	[18,22]	4	0.035	0.25	0.25	10	0.15	3	120	220
S5	R1	[34,50]	6	0.03	0.35	0.35	10	0.2	2	160	200
	R2	[25,35]	8	0.03	0.35	0.35	10	0.2	3	160	150

**Table 3:** The operation cost of manufacturer.

Manufacturer	Material Type	*HC_jk_*	*PC_k_*	*CR_jk_*
M1	R1	2	5	0.8
	R2	1	6	0.7

**Table 4:** The demands of the raw materials in each period.

M1	Material Type	*t* = 1	*t* = 2	*t* = 3	*t* = 4	*t* = 5	*t* = 6	*t* = 7	*t* = 8	*t* = 9	*t* = 10
Demand	R1	682	713	708	708	689	714	692	655	699	721
	R2	510	548	553	553	501	524	551	580	559	577

**Table 5:** The inventory level of the manufacturer in each period.

M1	Material Type	*t* = 1	*t* = 2	*t* = 3	*t* = 4	*t* = 5	*t* = 6	*t* = 7	*t* = 8	*t* = 9	*t* = 10
Inventory level (End in *t* = *i*)	R1	137	143	142	142	138	143	139	131	140	145
	R2	153	165	166	166	151	158	166	174	167	174
Purchasing quantities	R1	682	576	565	566	547	576	549	516	568	681
	R2	510	395	388	387	335	373	393	414	385	410

**Table 6:** The optimal purchasing quantities of selected suppliers.

Supplier	Material Type	Purchasing Quantities									
		*t* = 1	*t* = 2	*t* = 3	*t* = 4	*t* = 5	*t* = 6	*t* = 7	*t* = 8	*t* = 9	*t* = 10
S1	R1	200	200	200	200	200	200	200	200	200	200
	R2	10	0	0	0	115	0	0	0	0	0
S2	R1	0	0	5	6	0	0	0	6	8	0
	R2	280	175	168	167	0	153	173	194	165	190
S3	R1	150	150	0	0	0	150	0	150	0	150
	R2	0	0	0	0	0	0	0	0	0	0
S4	R1	160	160	160	160	160	160	160	160	160	160
	R2	220	220	220	220	220	220	220	220	220	220
S5	R1	172	66	200	200	187	66	189	0	200	171
	R2	0	0	0	0	0	0	0	0	0	0
